# Editorial: Current Challenges in Modeling Cellular Metabolism

**DOI:** 10.3389/fbioe.2015.00193

**Published:** 2015-11-26

**Authors:** Daniel Machado, Kai H. Zhuang, Nikolaus Sonnenschein, Markus J. Herrgård

**Affiliations:** ^1^Centre of Biological Engineering, University of Minho, Braga, Portugal; ^2^The Novo Nordisk Foundation Center for Biosustainability, Technical University of Denmark, Hørsholm, Denmark

**Keywords:** metabolism, modeling formalisms, metabolic networks, genome-scale modeling, kinetic modeling

Metabolism is a core process of every cell providing the energy and building blocks for all other biological processes. Mathematical models and computational tools have become essential for unraveling the complexity of cellular metabolism (Heinemann and Sauer, 2010). Models integrate current knowledge on a biological system in an unambiguous manner and allow simulating cellular responses to genetic and environmental perturbations. Advances in genome sequencing and annotation have facilitated the reconstruction of genome-scale metabolic models for hundreds of organisms, which are currently used in various applications ranging from human health to industrial biotechnology (Bordbar et al., 2014).

Despite these advancements, there are still major challenges in modeling cellular metabolism at the genome scale. These include the reconciliation of different modeling approaches, the integration of metabolic models with models of other biological processes, the interpretation of heterogeneous data sources using models, and the adoption of suitable standards for model sharing. The aim of this Research Topic is to present state-of-the-art methods that aim to overcome these challenges and push this frontier to a new edge.

Starting from the most fundamental aspect of biochemical reactions, Cannon (2014) reviews the historical perspective of thermodynamics as a major driving force in the evolution of life and presents a primer on statistical thermodynamics. The author then provides examples of thermodynamic analysis of small metabolic pathways, highlighting future directions for integration of thermodynamics and large-scale modeling.

The most common approach to build a metabolic model is bottom-up reconstruction, where individual reactions for a given organism are identified (through genome annotation and literature data) and retrieved from biochemical databases. This approach is mostly limited by the current knowledge on enzymes with annotated functions. The alternative (termed top-down) approach is to infer the underlying network structure by reverse engineering of metabolome data. Çakir and Khatibipour (2014) compare these two approaches, reviewing available methods for both cases and providing pointers toward the reconciliation of these strategies.

Once a model is built, it can be used to simulate the metabolic phenotype under different conditions and subsequently compared with *in vivo* results for validation and refinement. Phenotype microarrays currently allow high-throughput assessment of metabolic responses to multiple experimental conditions. Chaiboonchoe et al. (2014) present an optimization of the Biolog phenotyping protocol for metabolic profiling of microalgae. The experimental results are used to expand and refine a genome-scale model of the alga *Chlamydomonas reinhardtii* to include the utilization of carbon and nitrogen sources not present in the original model.

Choosing a modeling formalism requires a compromise between model size and detail (Machado et al., 2011). Constraint-based models have gained popularity for their scalability to the genome scale. However, when insight of intracellular dynamics is required, kinetic models become the obvious choice. Petri nets, with their varied extensions, offer an intermediate level of compromise, allowing structural network analysis and, to some extent, dynamic analysis. Hartmann and Schreiber (2015) present a unified graph formalism and implement transformation operations to convert from the unified model to any specific formalism. The authors provide an example of integrated analysis using different formalisms in a unified model of sucrose breakdown in the potato tuber.

Current *omics* technologies allow unprecedented quantification of different types of cellular components including RNA transcript, protein, and metabolite levels. Machado et al. (2015) use a multi-*omics* dataset of *Escherichia coli* to analyze the contribution of allosteric regulation in controlling central carbon metabolism. Given the role of this type of control in response to different perturbations, the authors present a new simulation method to account for allosteric interactions in the determination of steady-state flux distributions. This is the first constraint-based method to account for allosteric regulation.

Next-generation sequencing is another example of technology pushing the limits of biological discovery. Understanding how genetic variants affect metabolic phenotype is fundamental in diverse areas, such as the study of disease mechanisms and the engineering of microbial cell factories. Cardoso et al. (2015) review available methods to predict the effect of genetic variations in protein function and expression. Integrating these methods with genome-scale metabolic modeling creates the potential for mechanistically predicting the consequences of genetic variation in the cellular phenotype, which is currently not possible with the statistical approaches used in genome-wide association studies.

Microbial strain design is a common application of genome-scale models as the combinatorial explosion of possible genetic manipulations demands efficient optimization methods. Stanford et al. (2015) address the problem of butanol production in *E. coli* using a new strain design method, RobOKoD, that combines gene over/underexpression with gene knockouts, showing good agreement with experimental data. Khodayari et al. (2015) analyze the case of succinate overproduction in *E. coli* using k-OptForce, the first strain design method that accounts for integrated simulation of kinetic and constraint-based models. This enables strain design at the genome scale while accounting for regulation mechanisms in central carbon pathways, such as feedback inhibition. The authors observe decreased prediction accuracy when the kinetic model is applied in experimental conditions that differ from those used for parameter estimation, highlighting the importance of reparameterization of kinetic models for the conditions used in the production setting.

Last but not least, modeling the complexity of cellular metabolism is an iterative refinement process that cannot be accomplished without a community effort. The ability to share models using suitable standards is of paramount importance (Ebrahim et al., 2015). Dräger and Palsson (2014) present a comprehensive review of standardization efforts in Systems Biology, including standards for model representation, model visualization, minimum information requirements, and suitable ontologies. This review also covers public model databases, conversion tools, simulation software, and standards for publication of simulation results. Adoption of these standards is essential to ensure reusability of models and reproducibility of results.

The work presented in this Research Topic addresses many of the current gaps in the field with innovative solutions. Closing these gaps provides a stepping stone for the challenges to come. The future of metabolic modeling already holds exciting opportunities with a new generation of models that include protein structures, gene expression pathways, and even whole-cell models (King et al., 2015).

## Author Contributions

All authors have read and revised the manuscript.

## Conflict of Interest Statement

The authors declare that the research was conducted in the absence of any commercial or financial relationships that could be construed as a potential conflict of interest.
